# The hidden costs of national lockdowns

**DOI:** 10.1016/j.lanepe.2021.100035

**Published:** 2021-01-13

**Authors:** Santiago Garcia, Timothy D. Henry

**Affiliations:** a Minneapolis Heart Institute Foundation. Minneapolis, MN USA; b The Carl and Edyth Lindner Center for Research and Education, The Christ Hospital, Cincinnati, OH USA

COVID-19 has dramatically impacted healthcare delivery around the world. As hospitals systems prepared for the actual or perceived onslaught of COVID-19+ patients, measures were implemented that effectively discouraged or restricted patient access to outpatient care, and diagnostic and therapeutic cardiac procedures deemed elective. Furthermore, many governments adopted national lockdowns aimed at reducing the spread of the disease. National or regional lockdowns varied in scope and application from country to country but invariably caused restrictions in movements of people, workers and services, causing massive disruptions and economic loss [1]. Unintended healthcare consequences of these directives have been documented [2], [3], [4], [5]. For example, a significant reduction in the number of patients presenting with acute myocardial infarction (AMI) and other cardiovascular emergencies has been reported in the United States and Europe [2,3]. Late presentations of AMI with mechanical complications and an increase in the number of out of hospital cardiac arrests have also been documented [4,5].

In this issue of the journal Van Belle et al. [6] report on the incidence of AMI, both ST-segment elevation (STEMI) and Non-ST-segment elevation (NSTEMI), in two French provinces unequally affected by the pandemic (Hauts-de-France, 3^rd^ most affected by the pandemic, and Pays-de-la-Loire, one of the least affected). Another important difference between these 2 provinces is their approach to educating the public. Hauts-de France conducted a province-wide educational campaign emphasizing the importance of appropriate use of healthcare services during the pandemic whereas Pays-de-la-Loire conducted no such campaign.

From January to mid-May 2020 both provinces witnessed a decline in MI incidence rates (≅ 8%). Both provinces saw their declines in MI rates exacerbate further (≅ 20%) during the French national lockdown (March 18–May 10) but Hauts-de-France province normalized their MI numbers, relative to 2019, faster than Pays-de-la-Loire despite the fact that the former had 4 times the number of COVID-19 deaths. When missing MIs are reported as a proportion of COVID-19 deaths, a surrogate marker of disease burden and healthcare system overload, the province least affected by the pandemic had a higher percentage of missing MIs relative to the 3^rd^ most affected province (36% versus 18%).

By comparing comprehensive data from 2 provinces in the same country with very different COVID-19 disease burden, including public educational approaches, the authors add granularity to our understanding of this important public health issue that can inform policymakers going forward, as most of the United States and Europe enter a second and third wave of the pandemic. Public fear of contracting COVID-19 in the hospital system, not disease burden or healthcare system overload, appears to be the main driver of the reduction in AMI presentations during the pandemic. In fact, dramatic decreases in emergency department volumes were also reported [7].

For those patients who did present to the hospital system with AMI during the pandemic, the higher in-hospital mortality for STEMI patients (9.1% in 2020 vs. 6.2% in 2019) despite access to the cardiac catheterization laboratory is troublesome, and likely multifactorial. Previous observations in the US have reported longer door-to-balloon times during the pandemic [8]. Another consideration is the combination of COVID-19 and AMI, which significantly increases short-term mortality as reported in the North American COVID-19 Myocardial Infarction (NACMI) Registry. The incidence of STEMI with co-existent COVID-19 infection is not reported in the present study but requires consideration.

Some limitations of the study are worth mentioning. The investigators only captured AMI patients who received invasive angiography and therefore any practice changes that might have occurred during the pandemic, such as a shift from mechanical to pharmacological, or no reperfusion, would have been missed. In China, such a shift led to delays in reperfusion, increased mortality and risk of heart failure [9] and is not recommended by Society of Cardiovascular Angiography and Intervention (SCAI) and The American College of Cardiology (ACC) or European Society of Cardiology (ESC) [10].

National lockdowns were disruptive in nature but efforts to educate the public and maintain timely access to emergency care were important to minimize morbidity and mortality for patients with cardiovascular disease. Lockdowns instituted during the first wave of the pandemic failed to emphasize to the public the differences between hospitals (essential service) and non-essential services such as bars, restaurants and gyms. Also, the recommendation to “self-quarantine” for 2 weeks when symptoms of COVID-19 were present (many indistinguishable from heart disease such as dyspnea and cough) likely led many patients to delay or forgo needed medical care. The SCAI “Seconds Still Count” national public relations campaign likely contributed to the ‘recovery’ of STEMI activations in the United States. For governments considering additional lockdowns, approaches that preserve essential healthcare services and expand telemedicine capabilities, to include rural and institutionalized communities, are needed to avoid repeating the mistakes of the past.

## Contributions

Santiago Garcia: writing of first draft of the manuscript.

Timothy D. Henry: Editing and critical revision of the manuscript.

## Declaration of Competing Interest

Dr. Henry has nothing to disclose. Dr. Garcia reports grants and personal fees from Edwards Life-sciences, grants and personal fees from Medtronic, grants and personal fees from Abbott Vascular, grants from Boston Scientific, outside the submitted work.
